# Chemical and Resistive Switching Properties of *Elaeodendron buchananii* Extract–Carboxymethyl Cellulose Composite: A Potential Active Layer for Biodegradable Memory Devices

**DOI:** 10.3390/polym16202949

**Published:** 2024-10-21

**Authors:** Zolile Wiseman Dlamini, Sreedevi Vallabhapurapu, Jennifer Nambooze, Anke Wilhelm, Elizabeth Erasmus, Refilwe Mogale, Marthinus Rudi Swart, Vijaya Srinivasu Vallabhapurapu, Bheki Mamba, Wendy Setlalentoa, Tebogo Sfiso Mahule, Vanessa de Oliveira Arnoldi Pellegrini, Shaun Cronje, Igor Polikarpov

**Affiliations:** 1 Department of Maths, Science and Technology Education, Central University of Technology, Bloemfontein 9300, South Africa; wsetlale@cut.ac.za; 2 School of Computing, University of South Africa, Florida Park 1710, South Africa; vallas@unisa.ac.za; 3 Chemistry Department, University of Free State, Nelson Mandela Drive, Bloemfontein 9300, South Africa; n.jennifer33@gmail.com (J.N.); wilhelma@ufs.ac.za (A.W.); erasmuse@ufs.ac.za (E.E.); mogaler@ufs.ac.za (R.M.); swartmr@ufs.ac.za (M.R.S.); 4 Rand Water, Chemistry Department, Scientific Services Division, Vereeniging 1939, South Africa; 5 Physics Department, University of South Africa, 28 Pioneer Avenue, Florida Park 1710, South Africa; vallavs@unisa.ac.za (V.S.V.); mahults@unisa.ac.za (T.S.M.); 6 Institute for Nanotechnology and Water Sustainability, University of South Africa, 28 Pioneer Avenue, Florida Park 1710, South Africa; mambabb@unisa.ac.za; 7 São Carlos Institute of Physics, University of São Paulo, Jardim Santa Angelina, São Carlos 13560-000, São Paulo, Brazil; varnoldi@yahoo.com.br (V.d.O.A.P.); ipolikarpov@ifsc.usp.br (I.P.); 8 Physics Department, University of Free State, Nelson Mandela Drive, Bloemfontein 9300, South Africa; cronjes@ufs.ac.za

**Keywords:** resistive switching memory, NaCMC, plant extract, biodegradable

## Abstract

Biodegradable electronic devices play a crucial role in addressing the escalating issue of electronic waste accumulation, which poses significant environmental threats. In this study, we explore the utilization of a methanol-based extract of the *Elaeodendron buchananii* plant blended with a carboxymethyl cellulose biopolymer to produce a biodegradable and environmentally friendly functional material for a resistive switching memory system using silver and tungsten electrodes. Our analyses revealed that these two materials chemically interact to generate a perfect composite with near semiconducting optical bandgap (4.01 eV). The resultant device exhibits O-type memory behavior, with a low ON/OFF ratio, strong endurance (≥$10^{3}$ write/erase cycles), and satisfactory (≥$10^{3}$) data retention. Furthermore, through a comprehensive transport mechanism analysis, we observed the formation of traps in the composite that significantly improved conduction in the device. In addition, we established that altering the voltage amplitude modifies the concentration of traps, leading to voltage amplitude-driven multiple resistance states. Overall, our findings underscore the potential of functionalizing polymers that can be functionalized by incorporating plant extracts, resulting in biodegradable and nonvolatile memory devices with promising performance metrics.

## 1. Introduction

Functional organic materials and composites have become increasingly important in enhancing the capabilities of modern electronics. Organic materials such as organic polymers and composites offer unique advantages such as flexibility, light weight, low environmental footprint, adjustable conductivity, and low cost [1]. These characteristics make them ideal for the advancement of modern electronics. The number of organic materials is vast, and not all of them are suitable for use in electronics. However, organic materials with a conjugated π-system and a strong electron-acceptor or electron-donor group have more relevance for application in electronics due to their push–pull system (D–π–A) that enables efficient intermolecular charge transfer, resulting in low energy barriers [2]. Next-generation computer memory devices called resistive switching memories (ReRAMs or RRAMs) can be fabricated from such materials. In general, ReRAMs are fundamentally two-terminal devices with a metal–active layer–metal architecture. They can have at least two resistive states, which can be selected using an electrical stimulus. ReRAMs remains the most attractive emerging memory option compared to alternatives such as phase change memories (PCM), which is due to their excellent scalability (<10 nm), lower power consumption (sub-pJ),and high endurance (>$10^{12}$ write/erase cycles) [3]. Recent research has demonstrated that ReRAM cells can be easily assembled to create synapses in artificial neural networks [4]. This enables the development of neuromorphic systems that closely imitate the functioning of biological brains [5]. Furthermore, ReRAMs have been shown to be compatible with artificial intelligence and the Internet of Things [6]. In recent reports, Kim et al. recently fabricated a biodegradable and flexible agarose–gold nanoparticle composite-based ReRAM. This system not only showed large memory capacity $R_{ON}/R_{OFF}=10^{4}$ and endurance estimated at 10 years, but also an ability to emulate biological synapses with 64.4 pJ/event and 164.4 pJ/event potentiation and depression energy consumption, respectively [7]. This follows the observation of switching synaptic behaviour in ionic liquids [8].

In the present study, a functional biocomposite consisting of sodium carboxymethyl cellulose (NaCMC) blended with methanol-derived *Elaeodendron buchananii* plant extract (EBMeOH) was investigated for its resistive switching properties. Generally, CMC is a water-soluble derivative of cellulose, an abundant linear polysaccharide found in plants, algae, and the oomycetes. It is an anionic polymer decorated with $-CH_{2}COOH$ groups, and has found applications in industries such as food packaging, agriculture, drug delivery, water treatment, and more [9,10,11]. Cellulose can be derived from lignocellulose biomass, the most abundant biomass on Earth, with over 180 billion tons produced every year [12]. Of this amount, only 8.2 billion tons are currently used productively. The Brazilian agricultural industry alone generates about 600 million tons of residue per year. Considering that cellulose corresponds to some 35% to 45% of lignocellulosic biomass [13], this translates into 200 to 270 million tons of cellulose sustainably and renewably produced in Brazil every year. Furthermore, several reports have described sustainable carboxymethylcellulose (CMC) production from lignocellulosic residual biomass [14,15], allowing for sustainable production of CMC from the abundant residual lignocellulosic biomass. Vaporization of the residual lignocellulosic biomass opens up the possibility of building additional value chains and moving towards a circular and sustainable bioeconomy, particularly in developing countries. CMC and its composites have found utilization in various fields, including electronics. They have been utilized as electrolytes for energy storage devices [16], piezoresistive devices [17], sensing devices [18], and other applications [19,20]. When combined with graphene oxide, CMC composites have shown impressive results, with a large $R_{ON}/R_{OFF}$ ratio of $10^{5}$ achieved at 2.22 V [21]. The biodegradability and non-toxicity of CMC are well documented [22].

*Elaeodendron buchananii* (Loes.) is an evergreen shrub found in eastern Africa, specifically in Kenya and Uganda, and is used in conventional medical practices [23]. *Elaeodendron buchananii* plant extract is known to contain terpenes with important antimicrobial properties [24]. While little is known about the biodegradability of *Elaeodendron buchananii* plant extract, its overall similarity to other plant extracts actively used as additives in biopolymers (e.g., as additives in biodegradable films and coatings for active food packaging) and low concentrations of the extract used for the composite production allow us to speculate that the composite under study is indeed biodegradable [25]. Ethyl acetate-based *Elaeodendron buchananii* (EBETOAC) extract has exhibited both memory and threshold resistive switching when placed between indium-doped tin oxide (ITO) and Ag electrodes [26]. Apart from this report, no other studies have covered the use of *Elaeodendron buchananii* extracts in electronic devices. However, a variety of plant-based materials, including Euforbia Cotinifolia plant extract [27], Aloe Vera [28], pectin from orange peels [29] and banana peels [30], and many more. As far as we can tell, there has been no report to date of a polymer blended with a plant extract for ReRAM applications. Therefore, we report for the first time a functional NaCMC and EBMeOH composite used as a switching layer in a ReRAM device.

## 2. Experimental

### 2.1. Materials and Methods

#### 2.1.1. Collection of Plant Material

**Figure 1:**
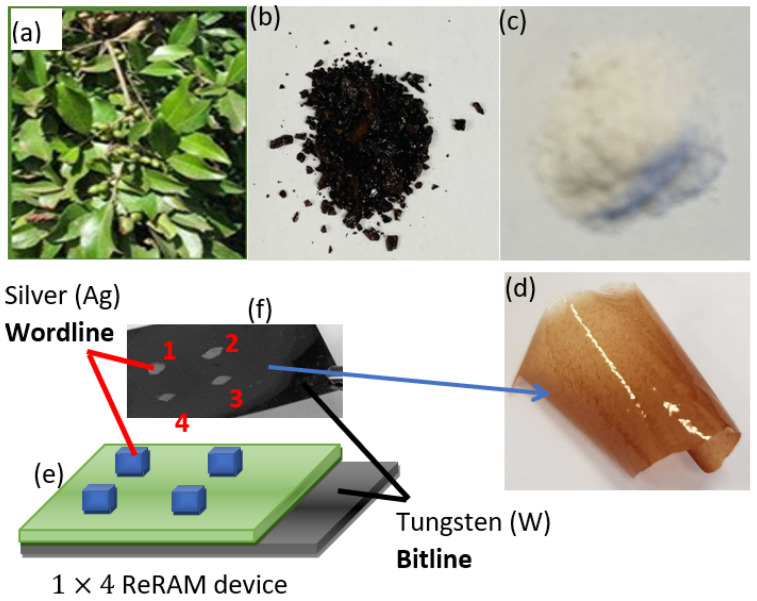
Photographs of (**a**) the *Elaeodendron buchananii* trunk, (**b**) the EBMeOH extract, (**c**) the Nacmc powder, and (**d**) the free-standing ABMeOH-NaCMC film; (**e**,**f**) show a schematic design and image of a 1×4 Ag/EBMeOH-NaCMC/W ReRAM, respectively.

The stem bark (Figure 1a) was obtained in September 2002 from Mabira woodland in Buikwe District, Uganda, situated between the districts of Jinja and Lugazi. Mr. Joseph Kavuma, a taxonomist, conducted species identification in the Makerere University Herbarium. This herbarium is managed by the Department of Plant Science, Microbiology, and Biotechnology, which is part of the College of Natural Sciences at Makerere University. The Department of Plant Science, Microbiology, and Biotechnology at Makerere University Herbarium received a voucher specimen of *Elaeodendron buchananii*, which was assigned the identification number EBKGt789.

#### 2.1.2. Extraction of Plant Material

The plant material was cut into smaller fragments and allowed to air-dry for 30 days at room temperature. The powdered plant material (500 g) was consecutively extracted at room temperature for 72 h with hexane, dichloromethane, ethyl acetate, methanol, and water (2.5 L). The extracts were concentrated to dryness under vacuum at 40 °C after being filtered through cotton wool. The extracts were further dried in a desiccator and refrigerated in tightly closed bottles for further analysis.

Extraction results: Hexane, dichloromethane, ethyl acetate, methanol, and water were employed as extraction solvents to extract plant constituents from *Elaeodendron buchananii*. A yellow extract (1.54%) was obtained from the hexane, an orange extract (11.21%) from the dichloromethane, a brown extract (5.07%) from the ethyl acetate, a dark brown extract (29.52%) from the methanol, and a dark brown extract (22.14%) from the water upon concentration.

Tannin removal from the methanol extract: Solid-phase extraction was used to remove tannins from a dried methanol extract of *Elaeodendron buchananii* stem bark (referred to as EBMeOH in this study). A large column was charged with polyamide gel (150 g), to which the reconstituted methanol extract was added (60.27 g in 1 L of methanol). Continuous additions of methanol were made to the column until it was colorless, indicating that all the tannins from the extract had been eliminated. The eluent was concentrated to dryness under reduced pressure. The dried methanol extract (EBMeOH) (Figure 1b) was weighed and stored in the fridge for further analysis.

#### 2.1.3. ReRAM Device Fabrication

EBMeOH (Figure 1b) and NaCMC (Sigma-Aldrich, St. Louis, MO, USA) (Figure 1c) were dissolved in methanol to create two solutions with a weight percentage of 1%. Afterwards, the two solutions were combined and gently mixed to create a uniform EBMeOH-NaCMC composite solution. The tungsten (W) and polyethylene terephthalate coated with indium-doped tin oxide (PET-ITO) substrates underwent a meticulous cleaning process using sonication in isopropyl alcohol, methanol, and distilled water. A small quantity of EBMeOH-NaCMC solution was dropcasted onto both W and PET-ITO surfaces and allowed to air dry for 24 h at room temperature. Unfortunately, the dried film on ITO lifted off and formed a standing EBMeOH-NaCMC film (Figure 1d), making it impossible to prepare any device using this substrate. However, we successfully created a stable film with thickness estimated as 0.47 mm on the W substrate, which enabled us to continue with the device fabrication process. In order to complete the device, a layer of silver (Ag) paste (Sigma Aldrich) was applied onto the surface of the film, resulting in the creation of four top electrodes with dimensions of about 2.4 × 2.4 mm^2^. The device was left to air-dry for an additional 48 h at room temperature. The device was built using the Ag/EBMeOH-NaCMC/W configuration, with Ag serving as the top electrode (TE) and W as the bottom electrode (BE). The device in question featured a single column and four rows, with designations of B=1 and $W^{*}=1,2,3\;\&\;4$, where B and $W^{*}$ represent bitline and wordline, respectively (here, we use $W^{*}$ instead of W, as the symbol W has already been used earlier for tungsten). Therefore, the device can be represented as a 1 × 4 Ag/ABMeOH-NaCMC/W ReRAM device. Figure 1e,f depicts a schematic diagram and photograph of the 1 × 4 Ag/ABMeOH-NaCMC/W ReRAM device fabricated in this study.

### 2.2. Characterization Techniques

#### 2.2.1. ATR-FTIR and Thermal Analysis

The functional groups of the compounds were examined by attenuated total reflectance–Fourier transform infrared (ATR-FTIR) spectroscopy (Bruker Tensor 27 model) with OPUS v1.1 analysis software and fitted with a Pike single-bounce diamond ATR crystal. For thermal decomposition and melting point determination, approximately 5 mg of the sample was weighed with a heating rate of 10 °C/min. For thermogravimetric analyses (TGA), the samples were analyzed on a Mettler–Toledo thermogravimetric analyzer (TGA/SDTA851) under a nitrogen atmosphere, then the obtained thermograms were evaluated by STAR SW 8.10 software. Melting points were determined by differential scanning calorimetry (DSC). The analysis was performed using DSC 5000 Discovery series equipment. The data were analyzed with TRIOS software.

#### 2.2.2. X-Ray Photoelectron Spectroscopy, SEM/EDS and AFM

X-ray Photoelectron Spectroscopy (XPS) was used to analyze the elemental composition of the samples. A PHI 5000 Versaprobe system with a monochromatic AlK X-ray source (AlKα = 1486.6 eV at 50 μm, 12.5 W, and 15 kV energy (97 X-ray beam)) was used for measurements. A JEOL JSM-7800F field emission scanning electron microscopy (FE-SEM) equipped with an Oxford Aztec energy dispersive X-ray spectroscope was used to study morphology and elemental composition. The electron beam voltage was maintained at 5 kV.

#### 2.2.3. Electrical Characterization

In order to comprehend the memory behavior of the device, electrical characterization was performed utilizing a specialized memristor characterization device available in-house. Throughout the measurements, the memory behavior of an individual cell was investigated. The initial set of measurements was acquired utilizing the $W^{*}=1|B=1$ configuration, 1 bitline and 1 wordline were employed. Specifically, the W electrode was linked to the bitline address, while the Ag electrode was connected to the wordline address.

## 3. Results

### 3.1. Morphological Characterisation

Morphological studies using scanning electron microscopy (see Figures S1–S4 in the Supplementary Information) revealed no significant differences between EBMeOH, NaCMC, and EBMeOH-NaCMC film. The EDS measurements of EBMeOH, NaCMC, and EBMeOH-NaCMC film (see Figure S5a–c in the Supplementary Information) detected the presence of mostly carbon and oxygen. Trace amounts of potassium, chlorine, and magnesium were also observed in the EDS of EBMeOH. For NaCMC, as expected, a substantial amount of sodium was detected due to the incorporation of sodium ions into the structure of the NaCMC. The EDS of the EBMeOH-NaCMC film is representative of a combination of EBMeOH and NaCMC, with a considerable amount of sodium as well as trace amounts of potassium and chlorine. In addition, silicon was also detected. Iridium (Ir) was present in all three samples, as the samples were coated with Ir to improve conductivity and reduce charging. An AFM was further utilized to observe the topography of the EBMeOH-NaCMC film (see Figure S5d,e in the Supplementary Information). The results show a smooth surface, with peaks and valleys measuring 440 nm in height. The estimated average roughness over the scanned area of 10 × 10 μm^2^ was 48.72 nm. The line profile (Profile 1) (see Figure S5f in the Supplementary Information) shows an irregular surface variation. These findings are in line with the previously discussed SEM data.

#### 3.1.1. XPS

**Figure 2:**
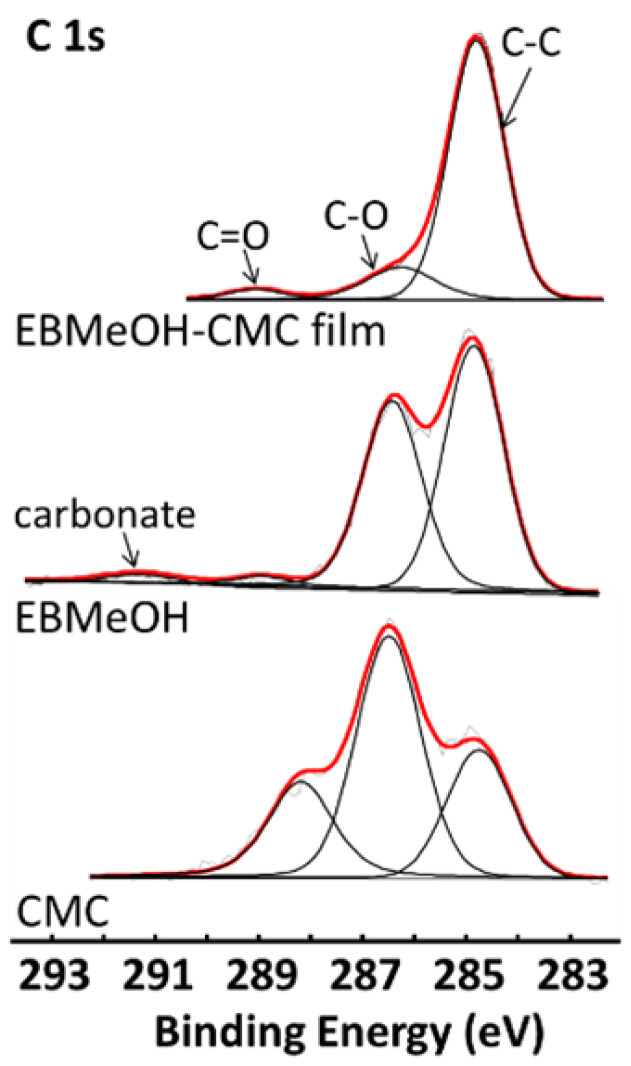
Detailed scans of the C 1s area of EBMeOH, NaCMC, and EBMeOH-NaCMC film. The red line represents the accumulated fitting of all the simulated peaks.

X-ray photoelectron spectroscopy (XPS) was used to probe the core electronic properties of EBMeOH, NaCMC, and EBMeOH-NaCMC films (see Figures S6–S8 in the Supplementary Information). The survey scans (presented in the Supplementary Information) revealed the presence of carbon and oxygen in all the samples, with the addition of sodium in the NaCMC samples, confirming the EDS results. The photoelectron lines (C 1s, O 1s and Na 1s) of the samples were deconvoluted with simulated peaks to obtain the smallest possible CHISquare value. The peak at the lowest binding energy of the C 1s photoelectron line, representing the C-C (as well as C-H and adventitious carbon), was set at 284.8 eV for all the samples to compensate for any charging. The other forms of carbon present in the samples were C-O and C=O (see Figure 2). EBMeOH showed an additional photoelectron line at around 291 eV; this was assigned to carbonate groups, which could be present in plant extracts. The data of the C 1s and Na 1s are summarised in Table 1. The binding energy position and shape of the photoelectron envelope of the CMC correspond very well with the results in reported the literature [31]. Because the binding energy (BE) of the C-O representing the ester and alcohol groups in the CMC, EBMeOH, and EBMeOH-CMC materials are in agreement, it can be deduced that the chemical environments of the C-=O carbons in all three samples are similar. However, the BE of the C=-O group of CMC present at 287.8 eV (belonging to the COO-Na^+^) is not present in the EBMeOH-CMC material. Because sodium ions are also absent in the XPS of EBMeOH-CMC materials, it can be assumed that the COO- from the CMC forms an interaction with the EBMeOH.

#### 3.1.2. ATR-FTIR

**Figure 3:**
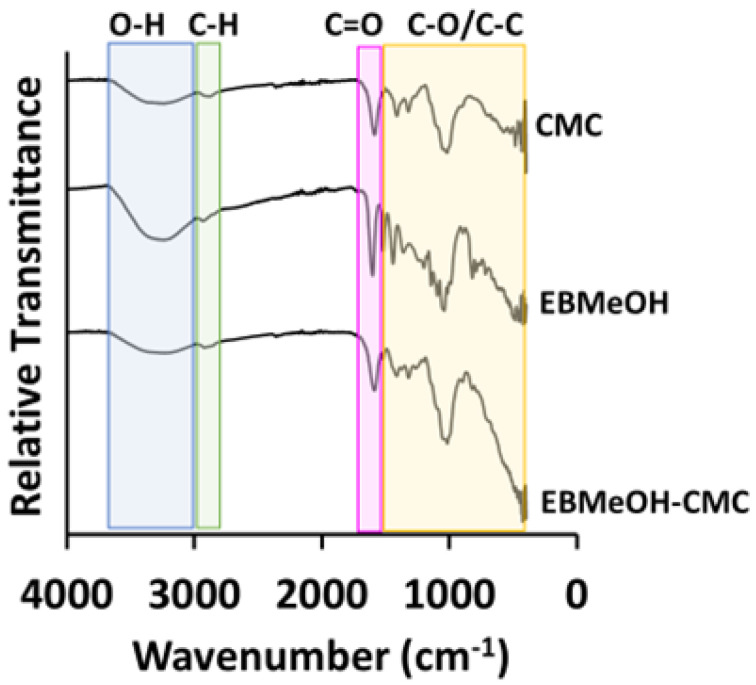
ATR-FTIR spectra of NaCMC, EBMeOH, and EBMeOH-NaCMC film composite.

The ATR-FTIR results are mutually consistent with the XPS results, confirming the presence of functional groups as well as potential linking (association) via the C-O group of the EBMeOH to the NaCMC. The ATR-FTIR of EBMeOH, NaCMC, and EBMeOH-NaCMC film are shown in Figure 3 and important data presented in Table 2. The symmetric and asymmetric stretching vibrations of the carboxylate groups (C=O) are represented by the bands at between 1400 and 1600 cm^−1^. The two small band at around 2921 and 2856 cm^−1^ correspond to C-H stretching. Within the range of 2900–3700 cm^−1^, a broad band is indicative of all the hydroxyl groups, attributed to the existence of both intermolecular and intramolecular hydrogen bonds. Considering that carboxymethyl cellulose is a glucose-type polymer, hydrogen bonding in both the intermolecular and intramolecular hydrogen bonds could potentially act as electron traps. In the fingerprint region, numerous sharp and intense peaks are observed, signifying C-O and C-C bending and stretching vibrations. The prominent band at 1018, 1087, and 1014 cm^−1^ (for NaCMC, EBMeOH, and EBMeOH-NaCMC, respectively) within the fingerprint region is attributed to the C-OH (primary alcohol group) stretching [32]. From the data in Table 2, there is a clear shift in the stretching frequencies of EBMeOH and EBMeOH-NaCMC film, which implies that a chemical association/bond is formed between the EBMeOH and NaCMC.

#### 3.1.3. NMR

**Figure 4:**
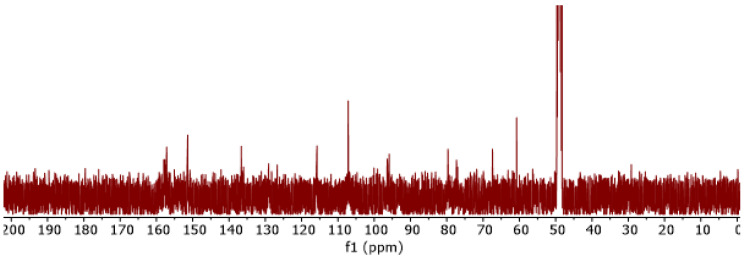
^13^*C* NMR spectrum of EBMeOH in MeOD.

**Figure 5:**
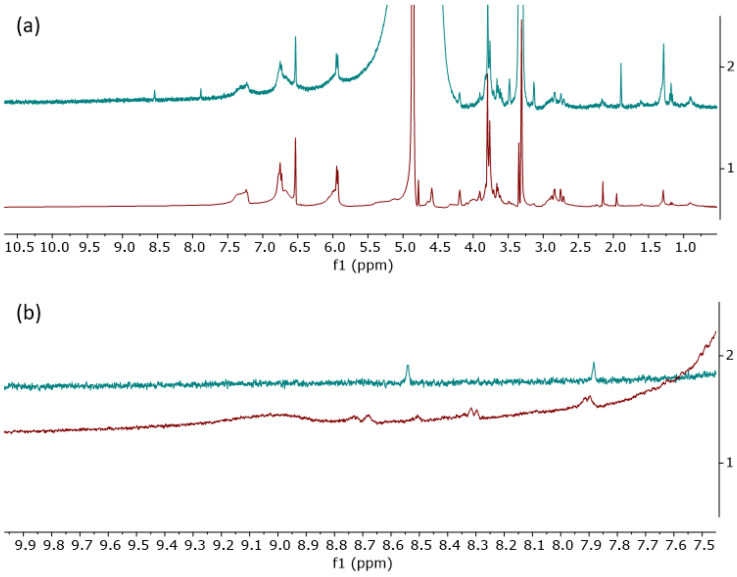
(**a**) The ^1^*H* NMR spectra of EBMeOH (1) and EBMeOH-NaCMC adduct (2) in MeOD; (**b**) expansion of the -OH region of the ^1^*H* NMR spectra for EBMeOH (1) and EBMeOH-NaCMC adduct (2) in MeOD.

^13^*C* nuclear nagnetic resonance (NMR) spectroscopy further supported the presence of -OH and =O groups in the EBMeOH sample, with resonances appearing in the carbonyl (δ150–180 ppm) and alcohol (δ50–80 ppm) regions, respectively (Figure 4). ^1^*H* NMR confirmed that the structural backbone of EBMeOH remained unchanged even after the addition of NaCMC (Figure 5a). However, changes in the -OH region (δ7.80–9.40 ppm) were observed after the addition of NaCMC, confirming potential bonding to NaCMC via the C-O group(s) of the EBMeOH (Figure 5b).

#### 3.1.4. Thermal Decomposition

The thermal decomposition and melting characteristics of the sample were assessed by means of TGA and DSC. The thermogram revealed a two-step decomposition process for all compounds, as outlined in Table 3 (see Figure S10 in the Supplementary Information). The initial decomposition occurring between 39 °C and 56 °C is attributed to the removal of volatile solvent molecules such as water and methanol. The complete structural decomposition for all samples takes place at temperatures exceeding 140 °C. Notably, pristine EBMeOH displays the lowest thermal stability, decomposing at 146 °C, while NaCMC exhibits the highest stability at 279 °C. The inclusion of NaCMC in the plant extract to produce the film results in significantly increased thermal stability of the composite. The decomposition temperature for the EBMeOH-NaCMC film is 187 °C.

#### 3.1.5. Optical Band Gap Energy

**Figure 6:**
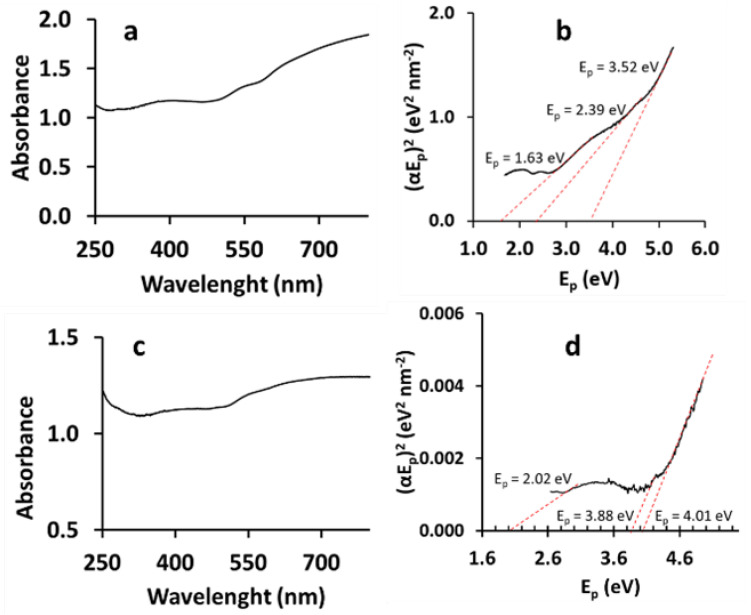
(**a**) The UV-Vis spectra and (**b**) Tauc plot indicating the optical band gap energy of EBMeOH. (**c**) The UV-Vis spectra and (**d**) Tauc plot indicating the optical band gap energy of EBMeOH-NaCMC film.

The solid-state UV-Vis absorption spectra of the EBMeOH and EBMeOH-CMC film (see Figure 6) were recorded to determine their optical band-gap energy at room temperature in the range of 250–800 nm. The optical band gap energies of EBMeOH and EBMeOH-NaCMC film were calculated from the absorbance vs. wavelength graphs (Figure 6) using Tauc’s equation (Equation (S1) in the Supplementary Information). This allows for the construction of a Tauc plot, which shows the relationship between the absorption coefficient and the optical band gap. The optical band gap of the EBMeOH and EBMeOH-NaCMC film were determined by analyzing the energy value where the line intersected the x-axis. This was done by extrapolating the linear portion of the curves, as shown in Figure 6 and in the data summarized in Table 4. The addition of NaCMC to EBMeOH resulted in a material with slightly elevated (=3.93 and 5.04 eV) optical band gap energies. This implies that the EBMeOH-NaCMC film needs more energy than pristine EBMeOH in order for the electrons to be excited, as the addition of NaCMC with its inherent wide bandgap results in increased optical bandgap.

### 3.2. Resistive Switching Characteristics

**Figure 7:**
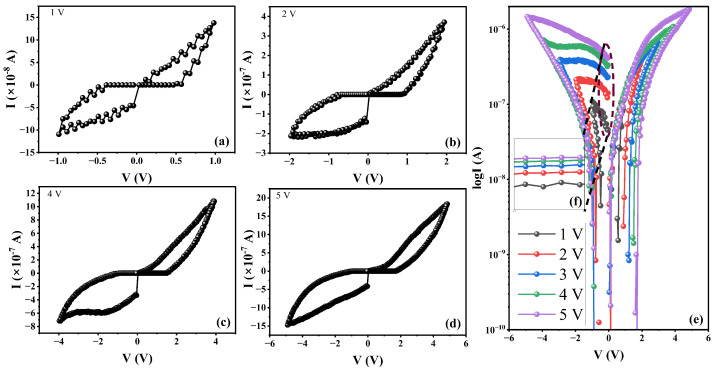
The complete set of I–V graphs for the Ag/EBMeOH-NaCMC/W device obtained at voltage amplitudes of 1 V (**a**), 2 V (**b**), 4 V (**c**), and 5 V (**d**) while employing a $W^{*}=1|B=1$ cell. Figure (**e**) depicts the amalgamated I–V graphs for the voltage scans ranging from 1 to 5 volts. (**f**) is the magnification of the graph segment marked with the dotted cycle.

The $W^{*}=1|B=1$ cell was thoroughly characterized by conducting a series of staircase increasing pulses. The initial voltage was set at 0.05 V, with an increment step of 0.05 V and a step width of 50 ms. The inter-pulse time was set at 10 ms, and a current compliance of 100 μA was used. The current–voltage (I–V) graphs in Figure 7 clearly demonstrate that the current takes different paths in the forward sweep and reverse sweep. This observation suggests that the $W^{*}=1|B=1$ cell possesses two distinct resistive states: a low-resistive ON-state and a high-resistive OFF-state. These resistive states are analogous to binary code, with “1” and “0” for the ON- and OFF-states, respectively [34]. The amplitude of the voltage cycle was increased from 1 V (Figure 7a) to 2 V (Figure 7b), 3 V, 4 V (Figure 7c), and 5 V (Figure 7d); the hysteresis with the relative best $R_{ON}/R_{OFF}$ ratio was observed at 4 V amplitude. In addition, during this increase in voltage scan amplitude, the device’s resistive states underwent shifts that resulted in the creation of wider hysteresis in the negative voltage bias (Figure 7e). This demonstrates a voltage amplitude-dependent multiple resistive state, as depicted in (Figure 7f). Multiple resistive states are an important characteristic feature for a device that can store multiple bits of data in a single cell. Voltage amplitude-induced multiple resistive states have been reported before in DNA molecule-based ReRAMs [35]. Not all systems exhibit multiple resistive states, and not all multiple states are induced by changing the voltage scan amplitude. In Ag/STO/Pt (where STO is $SrTiO_{3}$ and Pt is platinum), multiple resistive states were induced by changing the write voltage [36]. This was attributed to a change in the size of the conductive filament (CF). Changing the $I_{CC}$ is the most common way to induce multistate resistance, as reported for Ag/PMMA/(${BzA)}_{2}CuBr_{4}$/Pt [37], Ag/$CH_{3}NH_{3}PbI_{3}$/Pt [38], and other systems [39].

**Figure 8:**
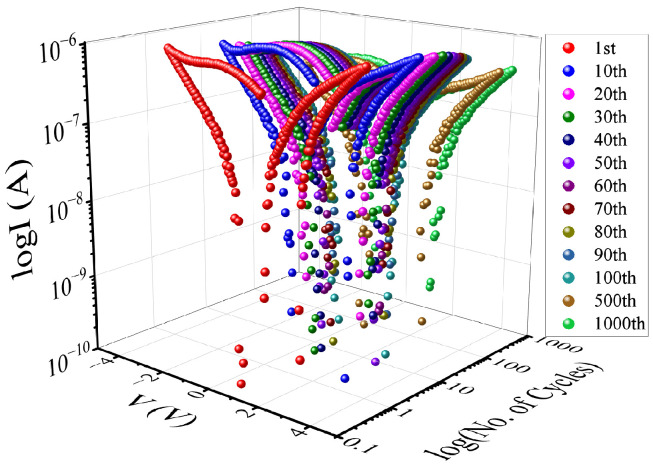
The I–V graphs for the Ag/EBMeOH-NaCMC/W device, with a voltage amplitude of 4 V, recorded for 1000 complete cycles. These measurements were taken specifically for the $W^{*}=1|B=1$ cell.

**Figure 9:**
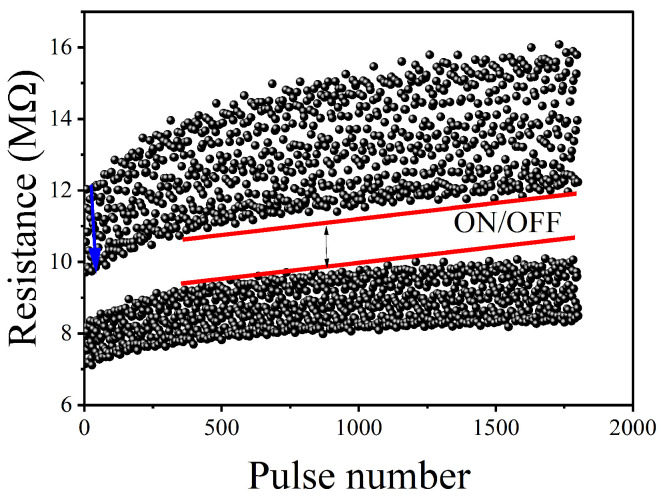
The endurance performance for the $W^{*}=1|B=1$ cell of the Ag/ABMeOH-NaCMC/W device measured during 1800 cycles.

**Figure 10:**
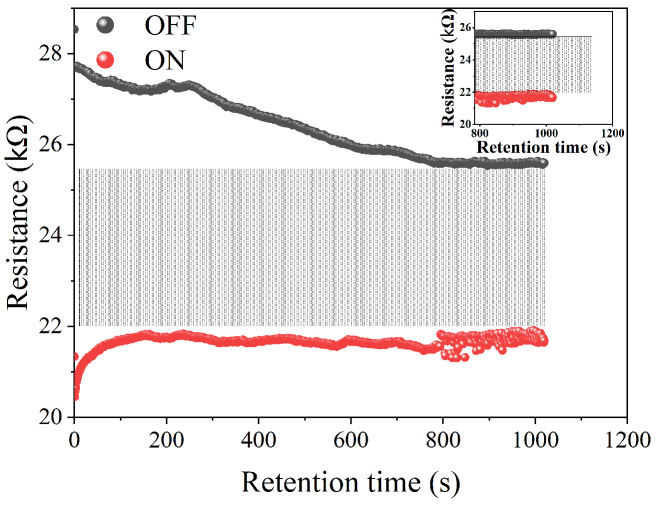
Data retention of LRS and HRS performed at 1 V as read voltage for the $W^{*}=1|B=1$ cell of the Ag/ABMeOH-NaCMC/W device.

A reliability study was performed by first running 1000 I–V scans at 4 V amplitude (Figure 8). Our data analysis shows that there was no significant change in I–V hysteresis, indicating that the device has good endurance. The durability of the $W^{*}=1|B=1$ cell was further examined by applying alternating ±1 V voltage pulses, each with a pulse width of 100 ms, to transition the cell from the OFF to the ON state and vice versa. This protocol emulates the write and erase protocols executed in a computer system. For this protocol, the readings were taken at a voltage of 0.5 V and with a current compliance of 100 μA. As depicted in Figure 9, our findings reveal clear resistive states, one in the OFF state and one in the ON state, with a resistance difference of approximately 2 megaohms. However, it is worth noting that there is considerable variation in the data for both states. Conducting a data retention study of the device was crucial to assess its suitability as a nonvolatile memory. The retention data shown in Figure 10 were obtained by applying a 1 V amplitude to select either the ON or OFF state. After the state was selected, the resistance of the cell was read every 1 s. Our data show that the resistance in both the ON and OFF states changes over time during the initial 800 s. After that, the resistance in both states remains constant, with some small data noise associated with the ON state. Nevertheless, our findings indicate that data retention over time is significantly improved, making this cell a promising option for non-volatile memory applications.

**Figure 11:**
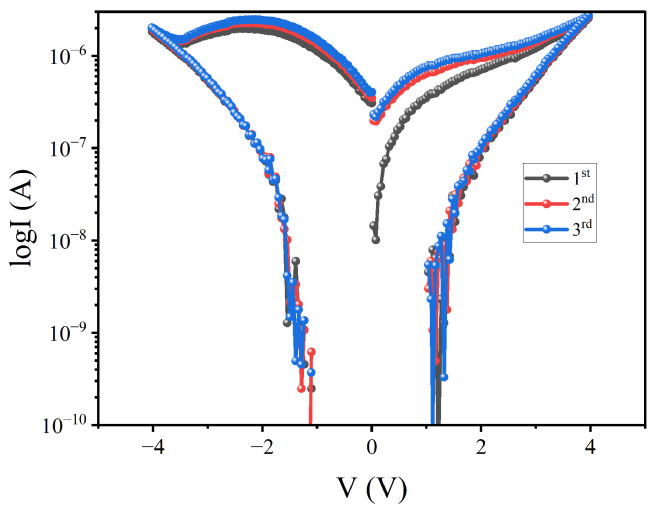
The I–V graphs for the Ag/ABMeOH-NaCMC/W device with a voltage amplitude of 4 V, recorded for three complete cycles. These measurements were taken specifically for a $W^{*}=4|B=1$ cell.

Finally, three additional memory cells were analyzed. The I–V data confirmed consistent memory behavior. The I–V variation of the $W^{*}=4|B=1$ cell for the first three voltage scan cycles is shown in Figure 11. Based on our data analysis, it is evident that every cell in this device exhibits memory behavior. Consequently, this device can serve as a reliable storage medium for computer data.

#### Transport and Switching Mechanism Analysis

**Figure 12:**
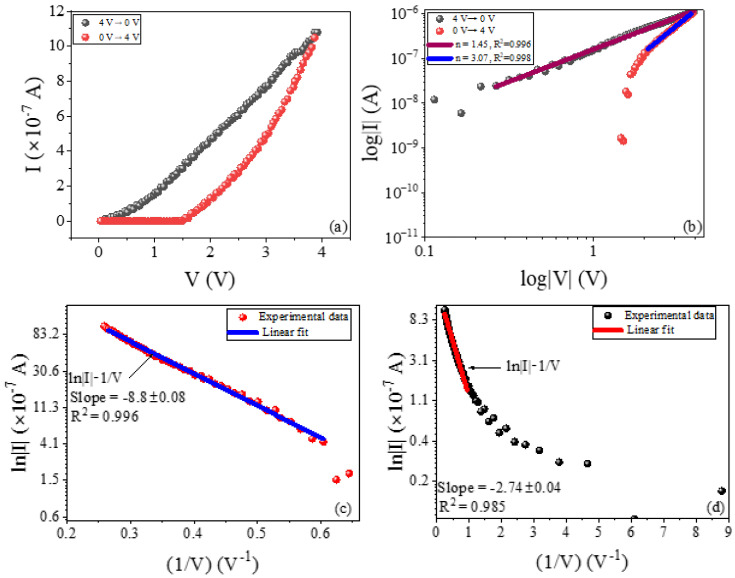
The (**a**) linear and (**b**) log–log I–V variation graphs along with the trap-assisted tunneling graph for the (**c**) OFF-state and (**d**) ON-state of the Ag/EBMeOH-NaCMC/W device.

Figure 12a–d shows the I–V variation of the device during the upwards (0 V → 4 V) (red) and downwards (4 V → 0 V) (black) voltage scans. From Figure 12a, it can be observed that the flow of electric current only begins after the voltage reaches the threshold of 1.5 V. This might be due to a high energy barrier at the electrode/composite interface, which must be overcome before electrons can cross the interface into the composite. This is a well-known Schottky barrier height ($\Phi_{B}$), which can be loosely estimated as the difference in work function between the Ag or W electrode and the EBMeOH-NaCMC composite’s electron affinity [40]. Schottky barrier effects have been reported previously in polymer composite-based ReRAM (Ag/PMMA@CsPbI3/FTO) devices [41]. Increasing the voltage reduces the barrier height, allowing electrons to finally penetrate the EBMeOH-NaCMC composite and resulting in a current, as illustrated Figure 12a. In this region, the I–V variation graph is not linear; instead, it resembles a modest exponential trajectory, indicating hopping-type conduction. However, further confirmation of this hypothesis is required. When the voltage is decreased, the current appears to follow a more linear trajectory than in the preceding scenario. To further explore these two graphs, we rescaled them to log–log scale, as shown in Figure 12b. By analyzing the slope of the log–log plot, we can determine the value of *n* for the relationship $J\propto V^{n}$ (where *J* is the current density and *V* is the applied voltage) to gain insights into the conduction mechanism in the composite. For the ON-state current, a linear fit was obtained with a value of n=1.45, which is greater than n=1 for the Ohmic case and less than n=2 for the Child’s square law case [42]. For the OFF-state current, the graph only shows a linear I–V relationship at high voltage, with n=3. After experimenting with various other mechanisms, the graph depicting the current in the OFF-state aligned perfectly with the trap-assisted tunneling (TAT) current model ($\ln J\propto -\frac{1}{V}$), as illustrated in Figure 12c. However, in the ON-state a linear region was only observed at higher voltage levels (Figure 12d). In these linear regions, the slopes were −8.8 A.$V^{-1}$ (with uncertainty of 0.08 A.$V^{-1}$) and −2.74 A.$V^{-1}$ (with uncertainty of 0.04 A.$V^{-1}$) for the OFF- and ON-states, respectively. The complete mathematical representation of the TAT conduction mechanism is as follows [42]:

(1) $$J_{TAT}=A\exp-\frac{8\pi \sqrt{2qm^{*}}}{3hE}\phi_{T}^{3/2}$$

where A is a constant, $\phi_{T}$ represents the trap energy level, which refers to the energy of the electron traps in relation to the conduction edge of the oxide, *E* denotes the applied electric field, $m^{*}$ stands for the electron effective mass in the active layer, and *h* represents Planck’s constant [43].

In order to explain the TAT mechanism in the system under investigation, we hypothesize that initially the electrons are unable to enter the EBMeOH-NaCMC composite due to the barrier energy at the Ag/EBMeOH-NaCMC and/or W/EBMeOH-NaCMC interface. As a result, no current is generated at low voltage. Because the EBMeOH-NaCMC system already contains oxygen, it is anticipated that this system will exhibit typical oxide film behavior, as described by Funck et al. [44]. Thus, as the voltage is raised, oxygen vacancies ($V_{O}^{2+}$) are generated and accumulate in the vicinity of the electrode. This reduces the energy barrier height at the interface, enabling electrons to penetrate the interface and resulting in the generation of current. Electrons are absorbed into the vacancies, which serve as trap sites in the Ag/EBMeOH-NaCMC/W system. This model’s signature is the re-emission of electrons from one trap site to the next, which can be attributed to various factors such as thermal effects. This phenomenon occurs until electrons successfully travel to the opposite electrode. When the voltage is decreased, the current takes a specific path, indicating a low level of resistance. This phenomenon occurs due to the relationship between the voltage and the number of defects. As the voltage increases, the number of defects also increases, resulting in exponential I–V behavior. Interestingly, when the voltage decreases, the system retains the previous state, characterized by a specific number of defects; hence, the system remains in a low-resistive state. According to this hypothesis, the behavior shown in Figure 7 proves that the voltage amplitude influences the concentration of defects, resulting in varying states of conductance and the observed multistate switching. We also acknowledge that as the W electrode is highly oxidizable, it may also contribute to the observed switching behavior.

## 4. Conclusions

EBMeOH, a methanol-based extract of the *Elaeodendron buchananii* plant, was blended with sodium carboxymethyl cellulose, a biopolymer, to create a biodegradable and ecofriendly functional material for resistive switching memory. Chemical and optical evaluation revealed that the two materials interacted, resulting in a composite with a larger optical band gap than either of the individual materials. This composite was tested as an active layer in a resistive switching memory device consisting of silver and tungsten electrodes. Electrical analysis of the entire device revealed significant current–voltage hysteresis typical of O-type memory behavior, revealing a low ON/OFF ratio but strong durability and fair data retention. Furthermore, we discovered through transport mechanism analysis that the development of traps in the composite enhanced conduction in the device and that changing the voltage amplitude altered the concentration of these traps, resulting in the voltage amplitude-driven multiple resistive states necessary for multi-bit data storage and neuromorphic computing. Overall, our findings demonstrate that polymers can be functionalized by combining them with plant extracts to create novel materials for biodegradable nonvolatile memory devices.

## Figures and Tables

**Table 1:** XPS data of the C 1s, O 1s, and Na 1s area along with the carbon assignment, binding energy (BE) of the peak maximum, and % present of the simulated fits.

	C 1s BE (eV)			Na 1s BE (eV)
Assignment	C-C	C-O	C=O	Na^+^
NaCMC	284.8	286.3	287.8	1071.7
EBMeOH	284.8	286.4	288.8	-
EBMeOH-NaCMC	284.8	286.3	288.9	-

**Table 2:** Stretching frequencies of the C-H, C=O, and C-O bands as measured by ATR-FTIR.

	C-H	C=O	C-O (Ester/Ether)	C-O (Primary Alcohol)
NaCMC	2910/2871	1585	1320	1018
EBMeOH	2933/2848	1600	1364	1087
EBMeOH-NaCMC	2919/2849	1584	1318	1014

**Table 3:** The decomposition temperatures of the different steps and melting points of EBMeOH, NaCMC, and EBMeOH-NaCMC film.

Material	Decomposition Temperature (°C)		Melting Point (°C)
	**Stel 1**	**Step 2**	
EBMeOH	39	146	111
NaCMC	56	279	121
EBMeOH-NaCMC	51	187	151

**Table 4:** The optical band gap energies of EBMeOH, NaCMC, and EBMeOH-NaCMC film (^*a*^ indicates data from [33]).

	Optical Bandgap Energy (eV)		
NaCMC	3.93 ^*a*^	5.04 ^*a*^	-
EBMeOH	3.52	2.39	1.63
EBMeOH-NaCMC	4.01	-	-

## Data Availability

The datasets used and/or analyzed during the current study are available from the corresponding author on reasonable request.

## Supplementary Materials

The following supporting information can be downloaded at https://www.mdpi.com/article/10.3390/polym16202949/s1, Figure S1. SEM images of (a) EBMeOH, (b) NaCMC, and (c) EBMeOH-NaCMC film at 60,000 times magnification; Figure S2. SEM images of EBMeOH at (a) 100, (b) 2000, (c) 6000, and (d) 30,000 times magnification; Figure S3. SEM images of NaCMC at (a) 100, (b) 2000, (c) 6000, and (d) 30,000 times magnification; Figure S4. SEM images of EBMeOH-NaCMC film at (a) 100, (b) 2000, (c) 6000, and (d) 30,000 times magnification; Figure S5. EDS spectra of (a) EBMeOH, (b) NaCMC and (c) EBMeOH-NaCMC. AFM topography image of the EBMeOH-NaCMC in (d) 3D and (e) 2D, and the (f) surface line profile of the EBMeOH-NaCMC film; Figure S6. XPS wide scan of NaCMC; Figure S7. XPS wide scan of EBMeOH; Figure S8. XPS wide scan of EBMeOH-NaCMC film; Figure S9. The thermal decomposition profile of (a) EBMeOH plant extract, (b) NaCMC, and (c) EBMeOH-NaCMC film; Figure S10. The melting point profile of (a) EBMeOH, (b) NaCMC, and (c) EBMeOH-NaCMC film.
