# Supplementary material for: Chemical and Resistive Switching Properties of Elaeodendron buchananii Extract–Carboxymethyl Cellulose Composite: A Potential Active Layer for Biodegradable Memory Devices
Source: Polymers (Basel). 2024 Oct 21;16(20):2949. doi: 10.3390/polym16202949 (PMC11511324; doi:10.3390/polym16202949)
Supplement: Supplementary file 1 [file polymers-16-02949-s001.zip › polymers-3211621-supplementary.pdf]

Article

Chemical and Resistive Switching Properties of *Elaeodendron Buchananii* Extract-Carboxymethyl Cellulose Composite: A Potential Active Layer for Biodegradable Memory Devices

Zolile Wiseman Dlamini [1*], Sreedevi Vallabhapurapu[2], Jennifer Nambooze[3], Anke Wilhelm[3], Elizabeth Erasmus[3], Refilwe Mogale[3,4], Marthinus Rudi Swart[3], Vijaya Srinivasu Vallabhapurapu[5], Bheki Mamba[6], Wendy Setlalentoa[1], Tebogo Sfiso Mahule[5], Vanessa de Oliveira Arnoldi Pellegrini[7], Shaun Cronje[8], and Igor Polikarpov6 [7]

1 Department of Maths, Science and Technology Education, Central University of Technology, Bloemfontein, 9300, Free State, South Africa1; zdlamini@cut.ac.za
2 School of Computing, University of South Africa, Florida Park, 1710, Gauteng, South Africa.
3 Chemistry Department, University of Free State, Nelson Mandela Drive, Bloemfontein, 9300, Free State, South Africa.
4 Rand Water, Chemistry Department, Scientific Services Division, Vereeniging, Gauteng, 1939, South Africa
5 Physics Department, University of South Africa, 28 Pioneer Avenue, Florida Park, 1710, Gauteng, South Africa
6 Institute for Nanotechnology and Water Sustainability, University of South Africa, 28 Pioneer Avenue, Florida Park, 1710, Gauteng, South Africa
7 S~ao Carlos Institute of Physics, University of S~ao Paulo, Jardim Santa Angelina, S~ao Carlos, 13560-000, S~ao Paulo, Brazil..
8 Physics Department, University of Free State, Nelson Mandela Drive, Bloemfontein, 9300, Free State, South Africa.
* Correspondence: zoliledlamini@hotmail.com

SUPPLEMENTARY INFORMATION

List of Figures

S1. SEM images of (a) EBMeOH, (b) NaCMC, and (c) EBMeOH-NaCMC film at 60 000 times magnification. . . . 3
S2. SEM images of EBMeOH at (a) 100, (b) 2000, (c) 6000, and (d) 30000 times magnification. . . . 3
S3. SEM images of NaCMC at (a) 100, (b) 2000, (c) 6000, and (d) 30000 times magnification. . . . 4
S4. SEM images of EBMeOH-NaCMC film at (a) 100, (b) 2000, (c) 6000, and (d) 30000 times magnification. . . . 4
S5. EDS spectra of (a) EBMeOH, (b) NaCMC and (c) EBMeOH-NaCMC. AFM topography image of the EBMeOH-NaCMC in (d) 3D and (e) 2D, and the surface line profile of the EBMeOH-NaCMC film. . . . (f). 5
S6. XPS wide scan of NaCMC. . . . 5
S7. XPS wide scan of EBMeOH. . . . 6
S8. XPS wide scan of EBMeOH-NaCMC film. . . . 6
S9. The thermal decomposition profile of a) EBMeOH plant extract, b) NaCMC, and c) EBMeOH-NaCMC film. . . . 6
S10. The melting point profile of (a) EBMeOH, (b) NaCMC, and (c) EBMeOH-NaCMC film. . . . 7

Equation S1: Tauc equation

$$(\alpha h\nu) = A(h\nu - E_g)^n \tag{S1}$$

where is an absorption coefficient, h is the photon's energy, A is the proportional constant, E_g is the band gap energy and n denote the nature of the sample transition. The value of n for allowed direct, allowed indirect, forbidden direct and forbidden indirect transitions are 0.5, 2, 3/2, and 3.31.

Equation S2: TAT equation:

$$J_{TAT} = A\exp\left(-\frac{8\sqrt{2qm^*}}{3hE}\phi_T^{\frac{3}{2}}\right) \tag{S2}$$

where A is a constant, ϕ_T represents the trap energy level, which refers to the energy of the electron traps in relation to the conduction edge of the oxide. E denotes the applied electric field, m stands for the electron effective mass in the active layer, and h represents the Plank's constant.

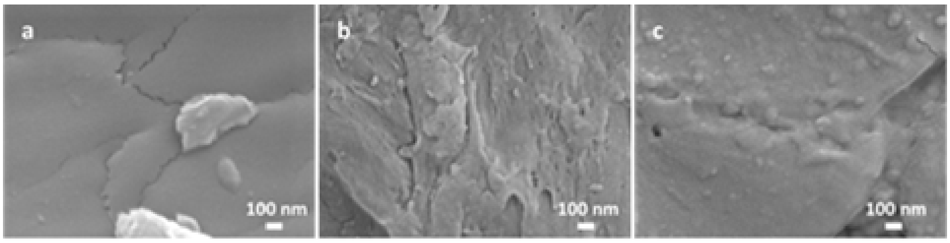

Figure S**1.** SEM images of (a) EBMeOH, (b) NaCMC, and (c) EBMeOH-NaCMC film at 60 000 times magnification.

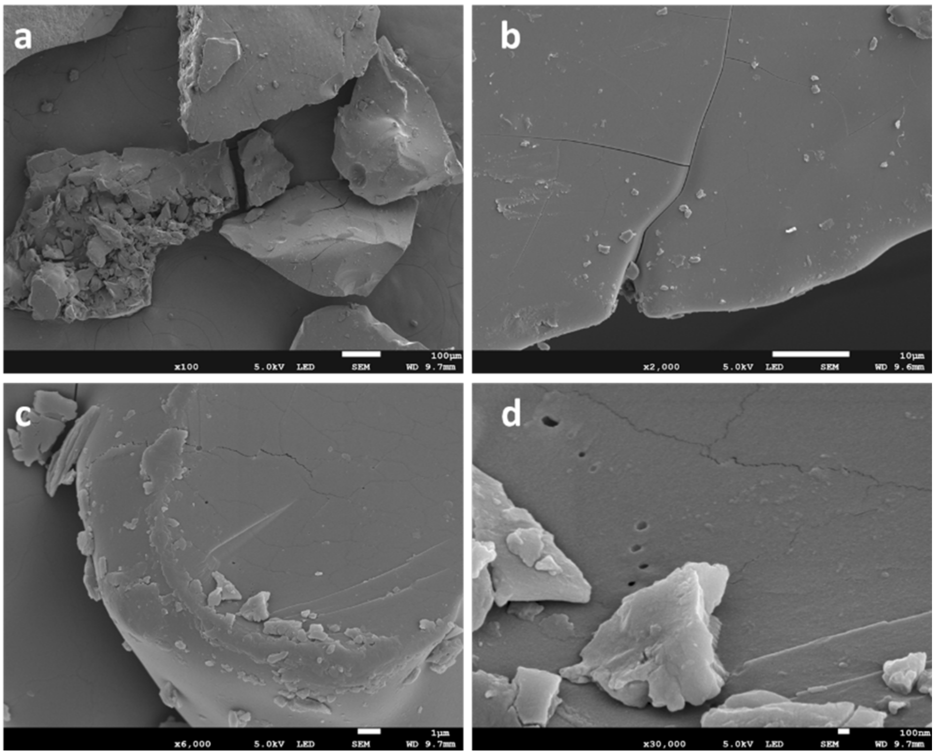

Figure S**2.** SEM images of EBMeOH at (a) 100, (b) 2000, (c) 6000, and (d) 30000 times magnification.

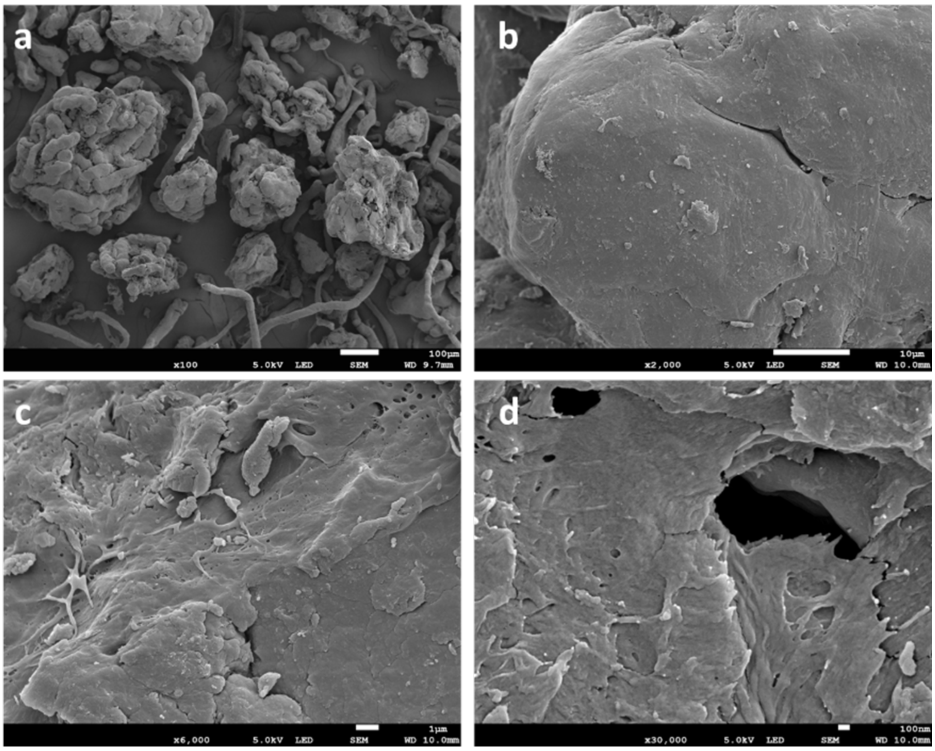

Figure S**3.** SEM images of NaCMC at (a) 100, (b) 2000, (c) 6000, and (d) 30000 times magnification.

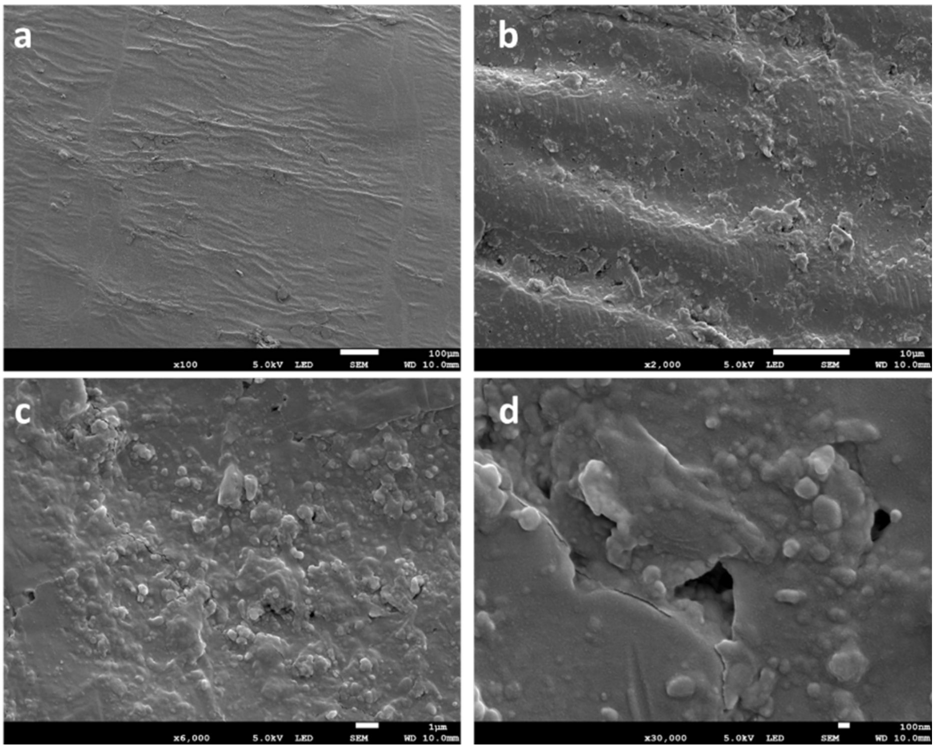

Figure S**4.** SEM images of EBMeOH-NaCMC film at (a) 100, (b) 2000, (c) 6000, and (d) 30000 times magnification.

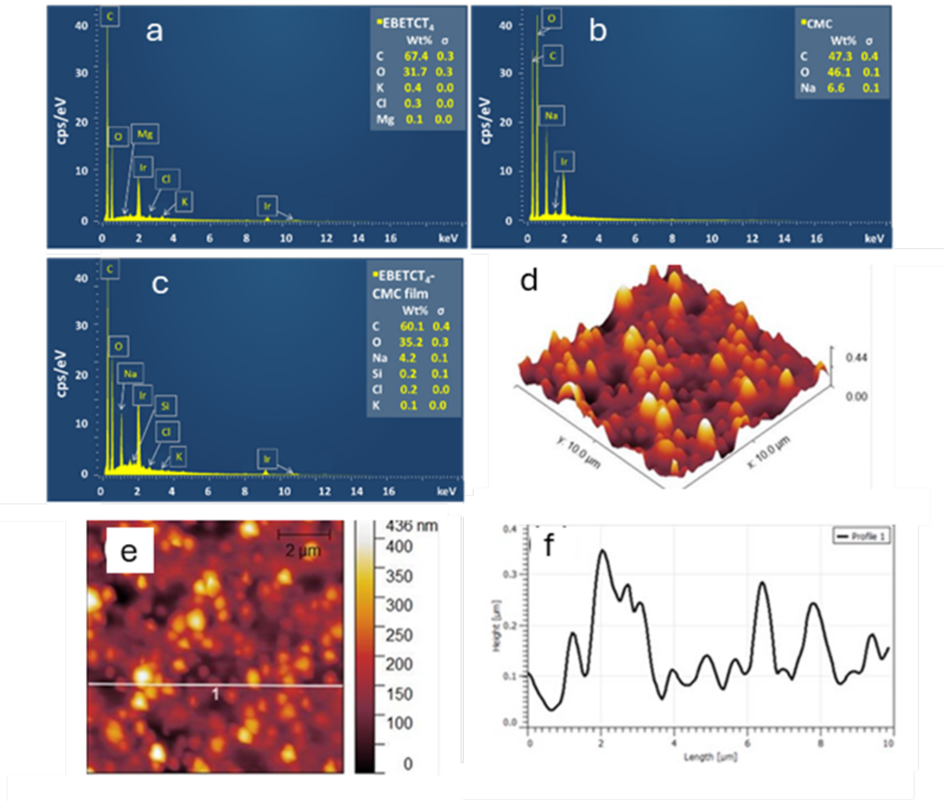

Figure S5. EDS spectra of (a) EBMeOH, (b) NaCMC and (c) EBMeOH-NaCMC. AFM topography image of the EBMeOH-NaCMC in (d) 3D and (e) 2D, and the (f) surface line profile of the EBMeOH-NaCMC film.

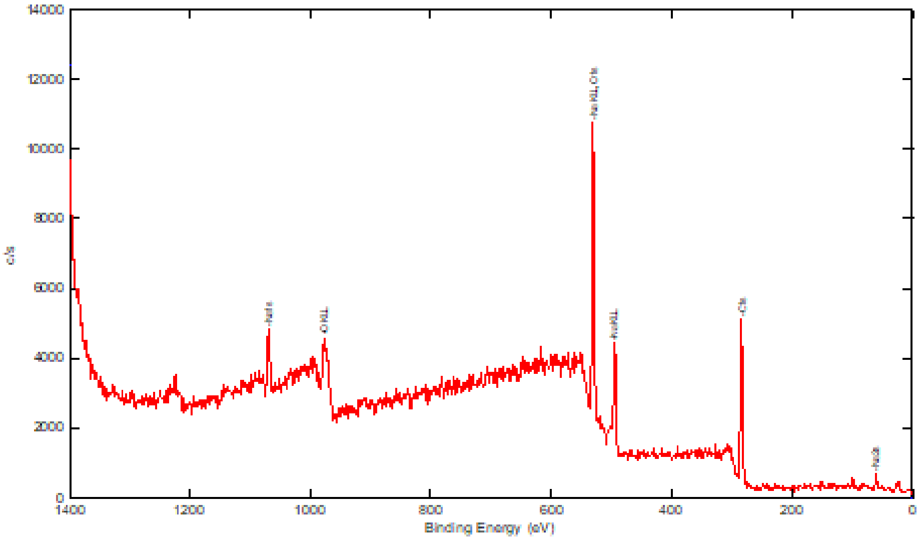

Figure S6. XPS wide scan of NaCMC.

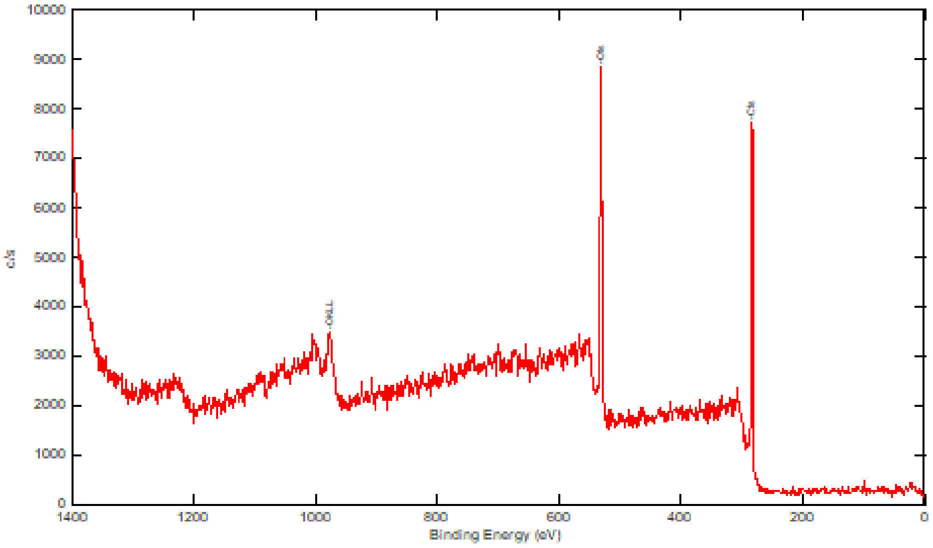

Figure S7. XPS wide scan of EBMeOH.

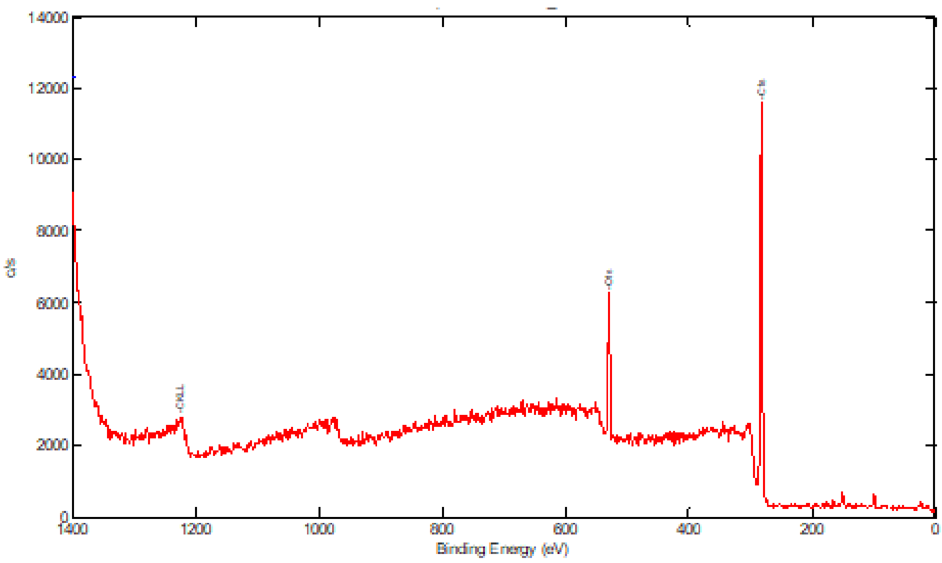

Figure S8. XPS wide scan of EBMeOH-NaCMC film.

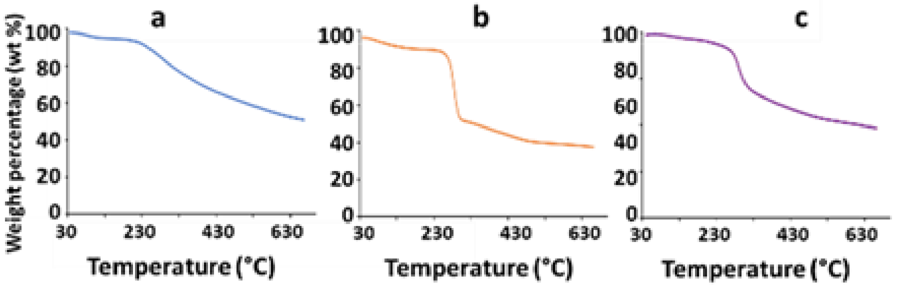

t

Figure S9. The thermal decomposition profile of a) EBMeOH plant extract, b) NaCMC, and c) EBMeOH-NaCMC film.

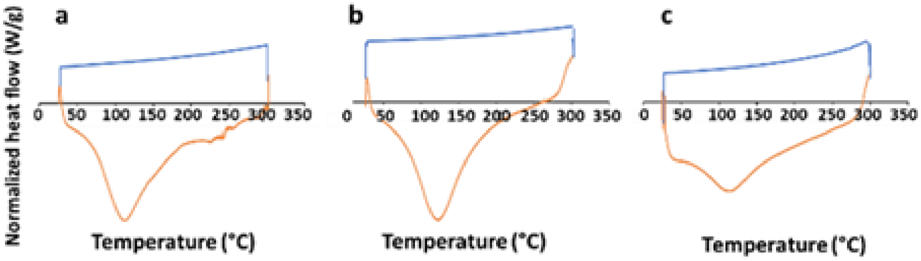

Figure S**10.** The melting point profile of (a) EBMeOH, (b) NaCMC, and (c) EBMeOH-NaCMC film.
